# Pediatric multiple sclerosis: Improving outcome through high-efficacy therapies

**DOI:** 10.1016/j.neurot.2025.e00631

**Published:** 2025-06-27

**Authors:** Lama Saleh Aljomah, E. Ann Yeh

**Affiliations:** Department of Pediatrics (Neurology), SickKids Research Institute, Division of Neurosciences and Mental Health, Hospital for Sick Children, University of Toronto, Toronto, Canada

**Keywords:** Pediatric-onset multiple sclerosis, Demyelinating disease, Disease-modifying therapy

## Abstract

Pediatric-onset multiple sclerosis (POMS) refers to multiple sclerosis occurring in individuals under 18 years of age. It is characterized by poor cognitive outcomes and a more inflammatory course, more frequent clinical relapses, and a greater number of MRI lesions compared to adult-onset MS (AOMS). Prompt recognition of multiple sclerosis in this population is essential, as early intervention with disease-modifying therapies may change the trajectory of disease progression. In this paper, we will review diagnostic criteria for pediatric multiple sclerosis, differential diagnosis, and current and emerging therapeutic approaches. While a number of DMTs are approved for adult MS, few are approved for pediatric use. Many of these DMTs are used off-label, with real-world evidence demonstrating their effectiveness and safety. The review evaluates existing evidence for the use of these therapies in pediatric populations, with an emphasis on both existing clinical trials and real-world data that supports their use. In addition, we will briefly highlight ongoing clinical trials and emerging therapies for POMS.

## Introduction

Pediatric-onset Multiple Sclerosis (POMS) accounts for approximately 3–10 ​% of all MS cases [1,2]. POMS differs significantly from adult-onset MS (AOMS) in several ways, including earlier disease onset, higher disease activity, distinct symptomatology, and a higher female-to-male ratio (2.8:1 versus 1.8:1 in adults) [3]. Diagnosing and managing POMS poses unique challenges due to the high frequency of MS mimics in this age group and the phenotypic variability of pediatric-acquired demyelinating syndromes (ADS) [[4], [5], [6]]. Moreover, given knowledge of high levels of disease activity and early disability in individuals with POMS, developing interventional strategies to reverse the course of the disease is of paramount importance.

Notably, few randomized controlled trials (RCT) of disease-modifying therapies (DMTs) have been performed in POMS, limiting the number of DMTs approved through formal regulatory processes for pediatric use. However, observational studies have demonstrated the associations between the use of high-efficacy disease-modifying therapies (HETs) and lower relapse rates, slower disability progression, and decreased MRI disease activity compared to low-to moderate-efficacy therapy (LMET), thus emphasizing the need for early access to HETs in POMS. In this paper, we highlight this work, starting first with a review of epidemiology, diagnosis and advances in imaging and biomarkers in POMS. We will then turn to a review of studies focused on therapeutic interventions for POMS and will focus specifically on evidence for early intervention and use of HETs in POMS. Finally, we will discuss non-pharmacological approaches, including diet and exercise, which may also contribute to improved outcomes in POMS.

## Epidemiology

Multiple sclerosis (MS) in individuals under 18 years of age is classified as pediatric-onset multiple sclerosis. It typically presents in mid-adolescence, with a mean age of onset of 14 years. Prepubertal onset (≤11 years) is rare, accounting for only 7.6 ​% of cases [3]. Sex differences vary with age. Before puberty, POMS affects males and females equally, whereas, after puberty females are 2–3 times more likely than males to develop MS [7,8].

The epidemiology of POMS varies regionally. MS occurs less frequently in youth than in adults, with a global annual incidence ranging from 0.05 to 2.85 per 100,000 individuals, and a pooled global incidence of 0.87 per 100,000. Published studies on incidence suggest higher rates in North America and Europe compared to Asia and the Middle East, although epidemiological studies are limited in some regions of the world [9]. Historically, MS has been described as predominantly affecting White females; however, evidence points to increased risk and rising incidence in non-White populations, including African American and Hispanic youth, in the United States and other regions [[10], [11], [12]].

The recognition of POMS has increased significantly over the past decade, potentially due to advances in MRI technology and refinement of diagnostic criteria. In 2013, approximately 7000 cases of POMS were reported across 34 countries, whereas by 2020, this number had increased to over 30,000 cases across 47 countries, representing a fourfold increase [13]. It is unclear whether this rise reflects a true increase in incidence or relates to enhanced diagnostic capabilities and greater awareness following the inclusion of pediatric-specific criteria in the 2017 McDonald Criteria for MS diagnosis [14].

## Clinical Presentation and Disease Course of Pediatric MS

### Clinical presentation

POMS typically presents with symptoms attributable to multiple neurological localizations, including optic neuritis, motor and sensory disturbances, and cerebellar dysfunction [1]. The most frequently reported presenting symptoms in children include optic neuritis (10–22 ​%), motor dysfunction (27–30 ​%), sensory symptoms (15–30 ​%), ataxia (5–15 ​%), and brainstem symptoms (22–41 ​%) [1,15,16]. The disease course in 96–98 ​% of POMS cases is relapsing-remitting (RRMS). Primary progressive MS is exceedingly rare in pediatric cases (<3 ​%) compared to adults (10–15 ​%) [17,18].

Children with POMS tend to experience more active disease with higher relapse rates and a shorter interval between the first and second demyelinating event when compared to individuals with AOMS. The annualized relapse rate (ARR) in POMS is 2.81 times higher than in AOMS in the first years following diagnosis even after adjusting for time on DMT, with persistence of the increased ARR (2.3:1) noted 6 years after onset [19,20]. Despite the higher disease burden, pediatric patients accrue disability more slowly and have better recovery from relapses than adults. Studies comparing AOMS and POMS found that POMS patients reached secondary progression later (median 16 years [4.34–44.33]) compared to adults (6.88 years [0.6–29]) and took approximately 10 years longer to develop irreversible disability [21,22].

### Cognitive and mental health outcomes

Approximately one-third of children with POMS experience cognitive impairment early on in the disease course: processing speed, executive function, and memory are most frequently affected [[23], [24], [25], [26], [27]]. A cross-sectional multicenter Italian study comparing 63 POMS patients to 57 healthy controls found that 31 ​% of POMS patients exhibited significant cognitive impairment. Domains which were most affected included memory, attention, verbal comprehension, and executive function, with younger age at onset predicting lower IQ scores (p ​= ​0.009) [28]. Evidence for worsening cognitive dysfunction can be seen in follow-up studies from this cohort: At two-year follow-up, 56/63 participants of the original POMS cohort were reassessed, with 70 ​% (39/56) classified as cognitively impaired. Three-quarters (39/52) demonstrated cognitive deterioration despite disease stability [29]. A five-year follow-up study of the same cohort (48/63 patients) confirmed cognitive deterioration in 56 ​% of patients. One fourth (12/48) showed improvement and 18.8 ​% (9/48) remained stable [30]. Most patients were treated with low-to moderate-efficacy DMTs, such as interferon-beta and glatiramer acetate. These studies are limited by selection bias: 15/63 (24 ​%) patients were lost to follow up through time. Nonetheless, these studies make the important observation that cognitive decline occurs early on in POMS, and that it is progressive. Long-term studies have identified the dynamics of change in cognition in POMS through time using the symbol digit modalities test (SDMT). Following improvements in SDMT scores for the first 10 years after onset, rapid declines in SDMT scores in POMS have been identified, with declines in POMS far greater than in AOMS [31].

Depression and fatigue are frequently seen in POMS and worsen through time [32]. Fatigue affects 20–43 ​% of POMS patients, while depression is reported in approximately one-third of cases [33,34]. Fatigue is linked to mental health conditions such as anxiety and depression, significantly impacting overall quality of life [35,36]. Furthermore, health-related quality of life (HRQoL) is significantly lower in children with MS compared to their healthy peers. Reduced HRQoL (Pediatric Quality of Life Inventory (PedsQL)) has been reported, with POMS participants (n ​= ​51) and their parents rating lower psychosocial and physical well-being than healthy age and sex-matched children [37]. Another study comparing children with MS (n ​= ​37) and clinically isolated syndrome (CIS) (n ​= ​13) to their siblings found that MS patients had a mean HRQoL score 16 points lower than their siblings (p ​= ​0.003) [38].

## Diagnosis of POMS

**Fig. 1:**
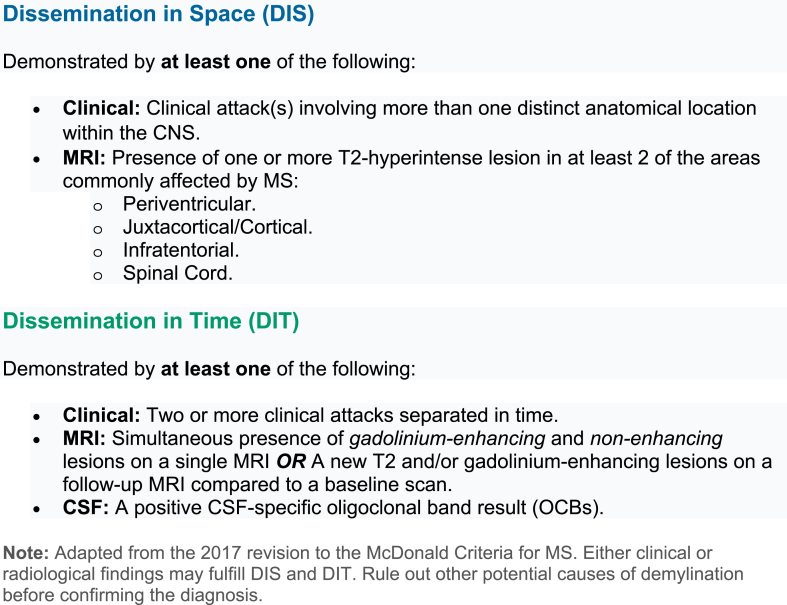
2017 Revised McDonald Criteria for Multiple Sclerosis.

According to the 2017 McDonald criteria for multiple sclerosis (Fig. 1), diagnosis of POMS can be made in the presence of clinical, MRI, or laboratory evidence of dissemination in time (DIT) and space (DIS), including the presence of oligoclonal bands in the CSF. Adult diagnostic criteria can be applied to children older than 12 years of age. Excluding alternative diagnoses remains crucial, especially in younger children, where demyelinating syndromes may mimic MS (discussed below, section 5). As such, younger children presenting with encephalopathy require a second episode to satisfy diagnostic criteria [14]. A prospective study of 324 children demonstrated 71 ​% sensitivity (95 ​% CI 56 ​%–83 ​%) and 95 ​% specificity (95 ​% CI 90 ​%–98 ​%) of the 2017 criteria for diagnosing POMS. MRI features including T1 hypointense lesions, gadolinium-enhancing lesions, and periventricular lesions improved differentiation between MS and monophasic demyelination in those patients [39]. Another prospective study evaluating 125 children with ADS found that 96 ​% of children diagnosed with MS met the 2017 McDonald criteria at presentation [40].

The McDonald criteria for MS diagnosis were revised in 2024: the criteria have not been published in final form yet [41]. The proposed revisions had the explicit aim of improving diagnostic accuracy and reducing delays by leveraging advancements in imaging and biomarkers and removing the mandatory requirement for DIT. Per revised criteria, diagnosis can be made based on DIS combined with the presence of oligoclonal bands (OCBs) or kappa-free light chains (kFLC), which have demonstrated equivalent or slightly improved value for diagnosis in MS when compared to OCBs [42]. Alternatively, an MS diagnosis can also be made through typical MS symptoms with characteristic lesions in at least four topographies Optic nerve lesions—identified using Magnetic resonance imaging (MRI), Optical Coherence Tomography (OCT), or Visual Evoked Potentials (VEP) – were included as a fifth MS topography. In addition, the new diagnostic criteria subsume select individuals with what was previously classified as radiologically isolated syndrome (RIS) under the MS umbrella. RIS is defined as the incidental discovery of CNS white matter T2-weighted hyperintense foci on brain MRI that demonstrate morphological and spatial characteristics highly typical of MS but without clinical symptomatology related to inflammatory demyelination [43]. The 2024 criteria allow for MS diagnosis if DIS criteria (lesions in 2 out of 5 topographies) are met in the presence of additional features including specific lesion characteristics (the presence of 6 lesions demonstrating the central vein sign (CVS)), one or more paramagnetic rim lesions (PRLs) or evidence of chronicity (DIT or oligoclonal band (OCB) positivity).

Inclusion of RIS in the new diagnostic criteria is of potential relevance to POMS, as studies have shown that children with MRI findings suggestive of demyelination have a 30–40 ​% risk of developing a new clinical event consistent with CNS demyelination within 2–3 years after presentation. These studies have identified factors such as the presence of ≥2 unique oligoclonal bands in CSF, spinal cord lesions, and callosal and juxtacortical lesions to be associated with an increased risk of developing a clinical event [44,45]. However, the ability to diagnose MS in asymptomatic children introduces important ethical and therapeutic dilemmas, particularly with regards to balancing the potential benefits of early intervention with the psychological impact and long-term treatment implications of a lifetime diagnosis for both the child and their family.

At present, information on whether the 2024 McDonald criteria will include a dedicated section on POMS is unavailable. However, the 2024 ECTRIMS presentation describing proposed changes to MS diagnostic criteria identified recommendations for MOG IgG testing in children under the age of 12 presenting with new CNS demyelination, and in children older than 12 years of age with atypical presentations [41]. It also remains uncertain whether the revised criteria will enhance the ability to make an early diagnosis of POMS, especially given the high sensitivity and specificity demonstrated by the 2017 criteria in this population [39]. As with prior iterations, validation studies will be essential to assess the applicability, diagnostic performance, and potential impact of the 2024 criteria in pediatric populations.

### Neuroimaging: Specific imaging findings

**Fig. 2:**
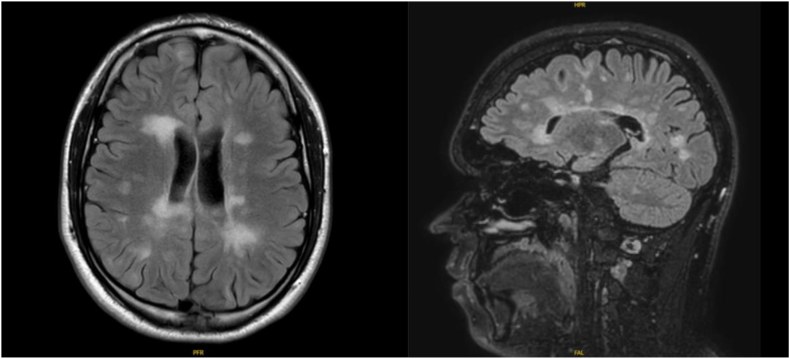
Axial and Sagittal FLAIR MRI scans of the brain demonstrating high lesion load at presentation in a 14 year-old child with MS. Note the presence of multiple discrete hyperintense lesions involving the supratentorial white matter and posterior fossa, involving the cerebellar peduncles and the cerebellar hemispheres bilaterally.

POMS is characterized by a higher total lesion burden early in the disease course compared to AOMS. Children with POMS exhibit greater T2-lesion count and volume (21 vs 6; *P* ​< ​0.001) and (4 vs 0; *P* ​< ​0.001) respectively, higher rates of gadolinium-enhancing lesions (68.4 ​% vs 21.2 ​%; *P* ​< ​0.001) and more infratentorial T2 lesions (68.3 ​% vs 31.4 ​%; *P* ​= ​0.001) than in AOMS [46,47]. Individuals with POMS have also been found to have higher T1-lesion volume (black holes) and an increased T1-lesion to T2-lesion volume ratio, together with greater gray and white matter loss after 20 years’ follow-up [48]. Moreover, longitudinal studies have demonstrated that children with MS experience abnormal brain growth trajectories, gray matter maturation, and thalamic growth compared to healthy children [49,50]. Additionally, higher lesion load has been found to correlate with greater thalamic volume loss over time in a longitudinal study of POMS (n ​= ​36) vs. healthy children (n ​= ​25) (p ​< ​0.0001) [51]**.** Whole-brain, gray matter, and white matter volumes at POMS diagnosis has been found to be significantly lower compared to age- and sex-matched healthy controls, with progressive brain atrophy noted through time, and correlations between higher lesion burden and greater brain volume loss at follow-up (p ​< ​0.001) [52]. Anatomical regions reported to be negatively impacted in POMS include frontal, temporal, parietal, and occipital lobes, as well as the cerebellum and caudate nucleus [[53], [54], [55]]. Fig. 2 illustrates the high lesion burden observed in POMS.

As for tools that may aid in MS diagnosis, specific MRI markers including the central vein sign (CVS) and paramagenetic rim lesions (PRL) can enhance diagnostic accuracy and aid in differentiating MS from its mimics. The presence of the CVS indicates perivenular inflammation in the white matter. Studies in adults comparing brain images from MS patients to those of individuals with other inflammatory conditions such as lupus report that the CVS has high sensitivity (up to 98 ​%) and specificity (94.4 ​%) for MS diagnosis [56]. Emerging evidence suggests that CVS improves diagnostic accuracy in POMS, although sensitivity (70 ​%) appears lower than in adults [57]. PRLs are another imaging modality that have been shown to have great utility in MS diagnosis in adults. PRLs indicate the presence of increased iron at the edges of lesions and are thought to represent perilesional chronic inflammation with iron-laden microglia/macrophages at the edges of MS lesions. Identification of at least one PRL has a specificity of 99.7 ​% in adult MS studies [58]. In POMS, 77 ​% of a cohort of 13 patients exhibited at least one PRL, with an average of one PRL per patient [59].

### CSF and serum diagnostic biomarkers

Cerebrospinal fluid (CSF) and serum biomarkers play an essential role in the early and accurate diagnosis of POMS. The 2017 McDonald criteria introduced OCBs as a proxy for dissemination in time (DIT). Although OCBs are used diagnostically as a substitute for dissemination in time in the 2017 and 2024 McDonald criteria, they do not themselves reflect the presence of a temporally distinct inflammatory event. Rather, their diagnostic utility stems from their strong association with future disease activity, thereby increasing the positive predictive value for MS diagnosis.

A study assessing the performance of the 2017 McDonald criteria in children with clinically isolated syndrome (CIS) found that diagnostic sensitivity increased from 46.8 ​% (2010 criteria) to 84.0 ​% with the inclusion of OCBs [60]. Furthermore, other studies have found that 96.1 ​% of POMS patients are OCB-positive compared to 90.0 ​% of adults (p ​= ​0.04), reinforcing the high prevalence and diagnostic utility of OCBs in POMS [61]. Age may make a difference in OCB positivity in the pediatric population. One study comparing children with earlier-onset MS (onset <11 years) to those with later-onset disease identified a significantly lower proportion of younger patients with an elevated IgG index (35 ​% vs. 68 ​%, p ​= ​0.031) [62]. Beyond aiding in diagnosis, OCB positivity also helps distinguish MS from monophasic demyelination. One study reported that 60 ​% of POMS cases had OCBs versus only 14 ​% of non-MS cases (HR 6.33; 95 ​% CI 3.35–11.96) [63]. It is important to note that these studies were conducted before the advent of MOG IgG testing, and it is not clear what proportion of the younger cohort might have been positive for MOG antibodies and therefore might be classified differently under current definitions.

Other biomarkers such as serum neurofilament light (NfL) and CSF kappa free light chains (kFLC) may offer additional diagnostic and prognostic value in POMS. Both reflect neural damage and chronic intrathecal inflammation and can be measured rapidly. CSF kFLC are 87 ​% concordant with OCBs in adult CIS cohorts [42,64], while in a cohort of 16 POMS patients a kFLC index >93.77 showed 68 ​% sensitivity and 100 ​% specificity for POMS [65]. Serum NfL (sNfL) correlates with disease activity and MRI lesion burden in POMS. A study of 61 POMS patients demonstrated that sNfL was 173 ​% higher in patients with active disease (relapse or new gadolinium-enhancing lesions within 60 days) compared to those who were clinically and radiographically stable. Additionally, patients treated with DMTs had 39.4 ​% lower sNfL levels than untreated patients (p ​= ​0.006), reinforcing its potential role in monitoring disease activity and treatment response [66]. A 2020 study found that sNfL levels in POMS decreased from 14.7 to 7.9 ​pg/mL after six months of effective treatment (p ​< ​0.001) [67].

## Differential Diagnosis

The clinical presentation of POMS poses significant diagnostic challenges, as its symptoms overlap with a number of pediatric demyelinating and neurological disorders. Accurate and timely differentiation is crucial to ensure appropriate treatment and avoid unnecessary interventions. Acute neurologic symptoms and white matter lesions on MRI, particularly in younger children, should prompt consideration of alternative diagnoses, including other acquired demyelinating syndromes (ADS) such as myelin oligodendrocyte glycoprotein antibody disease (MOGAD) and neuromyelitis optica spectrum disorder (NMOSD), as well as leukodystrophies, vasculopathies and mitochondrial disorders.

MOGAD is the most frequently occurring alternative diagnosis to MS in children. One study found that 18 ​% of a pediatric cohort of 65 patients who met the 2017 McDonald MS diagnostic criteria tested positive for serum MOG-IgG at initial presentation, suggesting the need for MOG antibody testing in children suspected of having MS. Importantly, this study identified atypical features in all children with MOG-antibody positivity. These features include age at onset of less than 11 years, diffuse, confluent bilateral lesions on MRI and complete or near complete resolution of initial MRI T2 lesions [68]. MOG-IgG testing via a cell-based assay is therefore strongly recommended in any child under 12 years presenting with a first-time demyelinating episode and in adolescents over 12 years if the presentation is atypical for MS.

Other considerations in the pediatric population include monogenetic disorders with a relapsing clinical course, such as primary CNS-hemophagocytic lymphohistiocytosis (HLH), leukodystrophies and mitochondrial disease. For instance, DNA polymerase gamma (POLG)-related disorder has been reported in patients presenting with optic neuritis, white matter hyperintensities and positive oligoclonal bands in the CSF mimicking MS [69,70]. Similarly, Familial hemophagocytic lymphohistiocytosis (fHLH) can also present with atypical chronic/recurrent CNS inflammation which may resemble MS in some cases. Given the low prevalence of primary progressive MS in children, a progressive disease course in a pediatric patient should raise suspicion for alternative diagnoses [71]. Finally, radiological features such as cortical lesions, symmetric deep gray matter lesions, posterior optic nerve/chiasm involvement and long segment spinal cord lesion should be evaluated carefully, as other disorders can often present with radiological features mimicking MS. Table 1 outlines key clinical and neuroimaging red flags that suggest alternative diagnoses.

**Table 1:** Differential diagnosis in pediatric-onset multiple sclerosis.

Clinical & Radiologic Clues	Differential Diagnosis
Encephalopathy, Polyfocal neurologic deficits; Diffuse, bilateral, large lesions, often affecting white and gray matter	MOGAD, CNS infections
Punctate (<3 ​mm diameter) deep-white matter T2-hyperintense Lesions and Psychosis or neuropsychiatric syndrome	SLE, vasculitis
Severe bilateral optic neuritis, longitudinally extensive optic nerve lesions extending to optic chiasm	AQP4+NMOSD, MOGAD
Bilateral disc edema with mild to moderate visual loss, vitritis	GFAP-IgG associated disorder, CRMP5 paraneoplastic optic neuropathy
Subacute vision loss with disc edema, uveitis. Linear T2-hyperintense lesions, Meningeal enhancement.	Neurosarcoidosis
Subacute vision loss with orbital disease	IgG4-related disease
Severe progressive optic neuritis or optic neuropathy (acuity of 20/200 or worse)	Leber hereditary optic neuropathy
Seizures, Extensive cortical lesions	MOGAD, autoimmune encephalitis, Vasculitis
Rapidly progressive disability	NMOSD, progressive multifocal leukoencephalopathy (PML)
Confluent T2-hyperintensities sparing U-fibers with developmental delay followed by progressive neurological decline	Neurodegenerative disorders such as leukodystrophies
Persistent symptoms like nausea, vomiting, vertigo, dysphagia. Extensive, confluent lesions, particularly in brainstem or spinal cord.	AQP4+NMOSD
Ophthalmoplegia, Large infiltrating brainstem lesions	Behçet's disease, Pontine glioma

1 ***AQP4+NMOSD*** aquaporin-4 antibody-associated neuromyelitis optica spectrum disorder, ***MOGAD*** myelin-oligodendrocyte glycoprotein antibody-associated disease, ***SLE*** Systemic lupus erythematosus.

## Pharmacologic Management of Pediatric-onset MS

### Management of acute relapses

Prospective trials on acute relapse management in children have not been performed, and most treatment protocols rely on historical data and expert consensus. Intravenous methylprednisolone (IVMP) at a dose of 30 ​mg/kg/day (maximum 1000 ​mg) for 3–5 days is generally used first-line in pediatric neuroinflammation [[72], [73], [74], [75]]. The Pediatric Optic Neuritis Prospective Outcomes Study (PON1) is a multicenter observational longitudinal study that evaluated visual recovery and disease progression in pediatric optic neuritis (ON). The study included 44 children who received corticosteroid treatment during their first ON episode. All children treated with corticosteroids demonstrated visual recovery within six months. Of the 28 participants who completed the two-year follow-up, 18 ​% experienced recurrent optic neuritis within that period, and all recurrent cases were later diagnosed with MS [76]. Oral prednisone equivalent dosing has also been utilized, with available evidence supporting its equal efficacy to IVMP, providing an alternative for outpatient management [77]. For patients with incomplete symptom resolution after steroids, intravenous immunoglobulin (2 ​g/kg divided over 2–5 days) is sometimes considered. However, class II studies have not demonstrated significant efficacy for IVIG in treating acute MS relapses, and its use remains controversial [78,79].

Therapeutic plasmapheresis (PLEX) or Therapeutic plasma exchange (TPE) is a widely used acute therapy in steroid-resistant fulminant demyelination with significant functional impairment, particularly tumefactive demyelination and in cases in which the brainstem is involved. Randomized sham-controlled studies in adult MS patients support the efficacy of PLEX in managing severe relapses, particularly in steroid-resistant optic neuritis (ON). The PLASMASEP trial, a double-blind RCT, demonstrated faster visual recovery in the PLEX group compared to the control group, with 50 ​% of ON patients achieving full recovery at 1 month compared to none in the sham treatment group. There were no differences in outcomes at 3 and 6 months, suggesting the benefit of PLEX primarily in time to recovery. The PLEX group showed a consistent functional benefit, as indicated by significantly better improvements in combined functional scores (CFS) of the Expanded Disability Status Scale (EDSS) at all follow-up points. While it did not include children, findings from this study could potentially apply to children [80,81].

A retrospective cohort study assessing the safety and outcomes of early PLEX administration in children with severe central nervous system (CNS) inflammatory demyelination included 12 pediatric patients with severe neurological impairment. PLEX was initiated within a median of 13 days after symptom onset due to either a lack of improvement (58 ​%) or worsening symptoms (25 ​%) despite corticosteroid treatment. At 3 months, 7 children (58 ​%) regained independent ambulation and showed functional improvement. Minor side effects (hypotension, hypersensitivity, and anemia) occurred in 3 patients, requiring intervention, but no life-threatening complications were reported [82].

### Relapse prevention: Disease-Modifying Therapy (DMT) use in POMS

MS Disease-modifying therapies (DMTs) are classified by their ability to reduce relapse rates, limit lesion formation, and slow disability progression in MS. Ocrelizumab, rituximab, ofatumumab, ublituximab, cladribine, natalizumab, and alemtuzumab are considered High-Efficacy Therapies (HETs). These therapies significantly suppress disease activity, but their use requires careful monitoring due to potential immunosuppressive risks, including infections and autoimmune complications. In contrast, low-to-moderate-efficacy therapies (LMETs) include agents such as interferon beta, teriflunomide, dimethyl fumarate, glatiramer acetate and fingolimod. They offer a safer profile but may be less effective in controlling disease activity and progression. A review of MS DMTs specifically used for POMS is provided below, including dosing, side effects, and monitoring requirements, and is summarized in Table 2. Table 3 summarizes ongoing clinical trials in POMS.

**Table 2:** Summary of the different disease-modifying therapies.

Medication	Dose	Common Side Effects	Monitoring Requirements
**Injectable medications**			
Interferon beta-1a	Avonex 30 mcg IM weekly Rebif 22 or 44 mcg SC 3 times a week	Flu-like symptoms, injection site reactions	Liver function tests, CBC
Interferon β-1b	Betaseron 0.25 ​mg SC every other day Extavia 0.25 ​mg SC every other day	Flu-like symptoms, seizures, injection site reactions	CBC, liver enzymes
Peginterferon β-1a	Plegridy 125 mcg IM/SC every 14 days	Flu-like symptoms, injection site reactions	CBC, liver enzymes, thyroid function
Glatiramer acetate	20 ​mg SC daily or 40 ​mg SC three times a week	Injection site reactions, chest pain	No routine monitoring required
**Oral medications**			
a^,^^b^Fingolimod	≤40 ​kg: 0.25 ​mg daily >40 ​kg: 0.5 ​mg daily	Bradycardia, macular edema, elevated liver enzymes	ECG before and after first dose, ophthalmologic exam. CBC and liver enzymes
bTeriflunomide	7–14 ​mg orally once daily	Alopecia, paresthesia, Gastrointestinal upset, liver toxicity	Screen for latent TB prior to initiation. Liver enzymes, blood pressure monitoring
bDimethyl fumarate	240 ​mg twice daily	Flushing, abdominal pain, diarrhea, nausea	CBC and liver enzymes
Cladribine	3.5 ​mg/kg over 2 years, followed by 1.75 ​mg/kg each year on 4–5 consecutive days for the first two months	Lymphopenia, infections, increased incidence of neoplasm in adult clinical trials.	Screening for latent TB, hepatitis B and C prior to initiation. CBC monitoring.
**Infusion medications**			
Ocrelizumab	Initial 2 doses: 300 ​mg; repeat dose 2 weeks later Subsequent doses: 600 ​mg every 6 months	Infusion reactions, increased risk of infections	Screening for hepatitis B, monitoring for infusion reactions
Rituximab	**Pediatric dosing:** 375 or 500 ​mg/m^2^ (max 1000 mg/dose) **Adult dosing:** 500 ​mg or 1000 ​mg. 2 infusions 2 weeks apart, then every 6 months. Extended interval dosing can be used. Reported interval median 18 months [112].	Infusion reactions, lymphopenia, hypogammaglobulinemia	CD19 every 6 months, CBC, liver enzymes, immunoglobulin levels
Natalizumab	300 ​mg every 4 weeks	Fatigue, diarrhea, urinary urgency/frequency, vaginal infections, dermatitis, PML	JCV antibody at baseline, then every 6 months (if negative) MRI brain every 3–6 months if high risk for PML
Alemtuzumab	12 ​mg IV daily for 5 days, followed by 12 ​mg daily for 3 days a year later	Infusion reactions, autoimmune conditions	CBC, thyroid function tests, renal function tests

2 ***CBC*** complete blood count, ***ECG*** electrocardiogram, ***JCV*** John Cunningham virus, ***PML*** progressive multifocal leukoencephalopathy, ***URTI*** upper respiratory tract infection, ***TB*** tuberculosis.

a Food and Drug Administration (FDA) approved for pediatric-onset multiple sclerosis.

b European Medicines Agency (EMA) approved for pediatric-onset multiple sclerosis.

**Table 3:** Ongoing clinical trials in pediatric MS.

Trial Name (NCT ID)	Medication	Type	Primary focus	Status
OPERETTA 1: NCT04075266	Ocrelizumab	Phase 2	Evaluate safety, tolerability, pharmacokinetics, and pharmacodynamic (PD) effects of ocrelizumab.	Active, not recruiting
OPERETTA 2: NCT05123703	Ocrelizumab	Phase 3	Evaluate safety and efficacy of ocrelizumab in comparison with fingolimod.	Active, not recruiting
CONNECT: NCT02283853	Dimethyl fumaric acid (BG00012)	Phase 3	Evaluate the safety, tolerability, and efficacy of BG00012.	Active, not recruiting
Peginterferonβ-1a: NCT03958877	Peginterferon Beta-1a (BIIB017)	Phase 3	Evaluate the safety, tolerability, and efficacy of BIIB017.	Active, not recruiting
NEOS: NCT04926818	Ofatumumab and Siponimod	Phase 3	Comparing the efficacy and safety of ofatumumab and Siponimod versus fingolimod.	Active, not recruiting

#### Low-medium efficacy therapies

1. Interferons

Interferon-beta (INF-β) has had long standing use in MS. While it is neither Food and Drug Administration (FDA) or European Medicines Agency (EMA) approved for POMS, a number of observational studies in POMS indicate that INF-β use is associated with a reduction in annualized relapse rates (ARR) and had few serious side effects [[83], [84], [85]]. These studies are limited by their retrospective nature and the lack of a comparison group, but nonetheless suggest safety and good tolerability. A 2013 multinational retrospective cohort study evaluated subcutaneous IFN-β1a in 307 children, reporting a 74 ​% reduction in relapse rate (ARR from 1.79 to 0.47). However, 32.2 ​% of patients discontinued IFN-β1a primarily due to clinical relapse (31 ​%), injection-site pain (2.9 ​%), flu-like illness (2.0 ​%), and liver enzyme abnormalities (1.3 ​%) [86]. INF-β reduces blood-brain barrier permeability and modulates T and B-cell function. Available formulations include interferon-β-1b, interferon-β-1a, and pegylated interferon-β-1a, with varying injection methods and frequency. In clinical practice, injectables have poor tolerability due to injection site reactions and flu-like symptoms which often limits adherence. Routine liver function and blood count monitoring are required due to potential cytopenia and liver enzyme elevations.

2. Glatiramer Acetate (Copaxone)

Glatiramer acetate (GA) is another injectable DMT. It is a synthetic amino acid polymer that mimics myelin basic protein and modulates T-cell responses toward an anti-inflammatory profile. Observational studies in POMS have shown that glatiramer acetate reduces relapse rates but does not significantly impact disability scores over a two-year follow up period [85,87]. These studies are limited by the lack of comparators and small number of patients (n ​= ​7, n ​= ​9 respectively). Its side effect profile is similar to INF-β but with milder injection site reactions and no requirement for routine laboratory monitoring. Glatiramer acetate-induced acute hepatotoxicity has been reported in one case of an adolescent with MS that developed significant hepatotoxicity within three months of starting GA, and which was reversible upon discontinuation of the therapy. Liver biopsy revealed hepatocyte necrosis, mitochondrial abnormalities and CD8^+^ T-cell infiltration, consistent with drug-induced hepatocellular injury [88].

3. Sphingosine-1-phosphate (S1P) receptor inhibitors

Fingolimod is a sphingosine-1-phosphate (S1P) receptor modulator which is EMA and FDA-approved oral DMT for POMS (ages 10 years and older). The main mechanism of action is S1P_1_ down-regulation which prevents lymphocyte egress from lymphoid tissues, thereby reducing auto aggressive lymphocyte infiltration into the central nervous system (CNS). The PARADIGMS phase 3 trial assessed the efficacy and safety of fingolimod compared to interferon beta-1a in POMS. It enrolled 215 patients (ages 10–17 years) who were randomly assigned to receive oral fingolimod (n ​= ​107) or intramuscular interferon β-1a (IFNβ-1a) (n ​= ​108), demonstrating an 82 ​% reduction in ARR (0.12 vs. 0.67) compared to interferon beta-1a. Additionally, there was a significant reduction in MRI lesion burden, with an annualized rate of new or newly enlarged lesions on T2-weighted MRI of 4.39 in the fingolimod group vs. 9.27 in the interferon beta-1a group (p ​< ​0.001). Overall, more patients remained relapse-free on fingolimod (85.7 ​%) compared to interferon beta-1a (38.8 ​%) at 24 months (P ​< ​0.001) [89]. Serious adverse events occurred in 16.8 ​% of fingolimod patients versus 6.5 ​% in the interferon beta-1a group, including bradycardia, macular edema, liver enzyme elevations, and seizures (5.6 ​% vs. 0.9 ​%) (P ​= ​0.006). Despite its higher rate of serious adverse events fingolimod was better tolerated than interferon beta-1a and only 7.5 ​% of fingolimod-treated patients discontinued the drug. Close monitoring, including ECGs, ophthalmologic exams, and liver function tests, is required. A follow up study in 2019 evaluating the predefined MRI outcomes from the PARADIGMS trial demonstrated (1) fewer T2 lesions for up to 24 months, (2) lower proportion of patients free of Gad ​+ ​T1 lesions, (3) lower number of Gad ​+ ​T1 lesions, and (4) lower volume of Gad ​+ ​T1 lesions in fingolimod vs. interferon-β-1a treated children [90].

Siponimod, a selective S1P receptor modulator approved for treatment for adult MS, is under investigation for POMS in clinical trials (NCT04926818). Other S1P receptor modulators such as ozanimod and ponesimod have been used in adults but have not yet been formally investigated in the pediatric population, and no clinical trials evaluating their use in children are currently being performed.

4. Teriflunomide (Aubagio)

Teriflunomide, approved by the EMA for POMS (ages 10 and older), works by inhibiting the proliferation of immune cells, specifically lymphocytes, by interfering with the DNA synthesis pathway. Teriflunomide was evaluated in the TERIKIDS trial in which 109 participants were randomized to oral teriflunomide and 57 to placebo for up to 96 weeks [91]. Because there was no significant difference in primary outcome (time to first clinical relapse) between the two groups, it was not approved by the FDA for use in POMS. It did have a favorable safety profile and efficacy in reducing MRI lesion burden, with a 55 ​% reduction in new/enlarging T2 lesions (p ​= ​0.00061) and a 75 ​% reduction in gadolinium-enhancing lesions (p ​< ​0.0001) in Teriflunomide compared to placebo. Importantly, there was a higher frequency of switching from the double-blind phase to the open-label treatment period due to high MRI activity, which may have reduced the power of the study. Two children treated with teriflunomide experienced pancreatitis, necessitating treatment discontinuation [91]. Other concerns include teratogenicity, alopecia, paresthesias, gastrointestinal upset as well as a black box warning for liver toxicity.

5. Dimethyl Fumarate (Tecfidera)

Dimethyl fumarate is an oral fumaric acid ester which activates the nuclear related factor 2 transcriptional (Nrf2) pathway. Dimethyl fumarate has received EMA but not FDA approval for POMS (>13 years). The safety profile and efficacy of dimethyl fumarate in youth with MS were first demonstrated in CONNECTED trial, a 96-week extension of the FOCUS phase 2 study that found decrease in ARR to 0.6 after 24 weeks on DMF that further dropped to 0.1 after 96 weeks [92,93]. The *CONNECT* study was an open-label, rater-blinded, 96-week randomized clinical trial comparing DMF (n ​= ​78) to interferon beta-1a (n ​= ​72) in children and adolescents with MS. The study found that a higher proportion of patients in the DMF group remained free of new or enlarging T2 lesions (16.1 ​%, 95 ​% CI: 8.0 ​%–27.7 ​%) compared to the interferon beta-1a group (4.9 ​%, 95 ​% CI: 0.6 ​%–16.5 ​%). However, a key limitation of the trial was the high dropout rate. Additionally, there was no significant difference in the adjusted annualized relapse rate, though the safety profile was comparable between groups [94]. Mild adverse events were observed and included gastrointestinal side effects and flushing. Two pediatric patients in the dimethyl fumarate group in the CONNECT trial developed lymphopenia, one of which was classified as severe, leading to discontinuation of the medication [94].

#### High efficacy therapies (HET)

1. Natalizumab

Natalizumab (NTZ) is an infusion-based DMT which inhibits the migration of inflammatory cells to the CNS by targeting the α4-integrin receptor. Several retrospective and prospective studies have demonstrated the safety and efficacy of natalizumab in POMS, with generally mild adverse events [[95], [96], [97], [98]]. An Italian registry study of 101 youth with MS who received natalizumab infusions demonstrated a decreased of ARR to 0.1 ​± ​0.3 during NTZ therapy compared to 2.3 ​± ​1.3 ARR before treatment (p ​< ​0.001). 58 ​% of patients achieved “No Evidence of Disease Activity” (NEDA), meaning they experienced no relapses, no disability worsening, and no new MRI lesions [95]. A retrospective multicenter study comparing natalizumab, fingolimod, and injectable medications found that patients in the natalizumab group had the highest proportion free of clinical relapse at years 1, 2, and 5 of treatment, and that the time to treatment discontinuation was significantly longer for natalizumab and fingolimod than for injectable DMTs (p ​< ​0.001). Unfortunately, there was limited data on safety and PML risk in natalizumab-treated patients in this paper [99].

The risk of progressive leukoencephalopathy (PML) increased with natalizumab, particularly when combined with a positive anti-John Cunningham virus (anti-JCV) status and prior treatment with other immunosuppressive agents [100]. There have been no reported cases of PML in POMS treated with natalizumab: an Italian registry study had no cases of PML after a mean treatment duration of 34.2 months despite 43 ​% of patients being positive for anti-JC virus (JCV) antibodies [95]. Regardless, given the potential risk of PML with natalizumab use, one must weigh risks and benefits of use of natalizumab in this population and monitor anti-JCV antibody status regularly in POMS. Additionally, its fixed monthly intravenous dosing schedule may pose logistical challenges for children and families.

2. B-cell depleting therapies

B-cell depleting therapies are used widely in MS, with multiple studies demonstrating effectiveness and efficacy in adults with MS of therapies including rituximab (anti CD-20 chimeric monoclonal antibody) ocrelizumab (anti-CD 20 humanized monoclonal antibody), ofatumamab (anti-CD 20 fully-humanized monoclonal antibody), and ublituximab (anti CD-20 chimeric monoclonal antibody) [[101], [102], [103], [104]]. Of these, 3 are FDA and EMA approved, and one, rituximab, is widely used off-label [105,106]. While no RCTs have been completed on these agents in the POMS population, clinical trials actively enrolling POMS patients include those evaluating ofatumumab (NCT04926818) and ocrelizumab (NCT05123703, NCT04075266). A multicenter retrospective study evaluating the use of rituximab in POMS (n ​= ​61), median follow up 20.6 months) showed a decrease in ARR from 0.60 (95 ​% CI 0.38–0.92) to 0.03 (95 ​% CI 0.02–0.14). Mild infusion-related reactions were seen in 48 ​% of patients [106].

With regards to ocrelizumab, in addition to an ongoing phase 3 clinical trial (NCT05123703) evaluating efficacy in POMS, the OPERETTA I study established pediatric-specific dosing (300 ​mg for <35 ​kg, 600 ​mg for ≥35 ​kg) every 24 weeks [107]. As for real-world data, a multicenter cohort study of 37 POMS patients treated with ocrelizumab (OCR) demonstrated a significant reduction in ARR from 1.08 to 0.08 (95 ​% CI: 0.00–1.57). By six months, 91 ​% of patients achieved NEDA-2 (no relapses, no new MRI activity), and 89 ​% maintained NEDA-2 at 12 months. No severe adverse events leading to treatment discontinuation were reported [108]. Another observational study of ocrelizumab in POMS (n ​= ​10, follow up ​> ​12 months) demonstrated a decrease in ARR (2.01 (±0.71) to 0) (p ​< ​0.0001), with no evidence for MRI activity in any of the patients in the follow-up period of 24.5 months (IQR:12–44 months). With regard to safety, one patient experienced grade 3 anaphylaxis during the first infusion but was able to continue OCR after desensitization. No serious opportunistic infections or clinically significant hypogammaglobulinemia were reported in this study, although two patients were found to have low IgM levels (<45 ​mg/dl). All patients had normal levels of IgG and IgA after a median follow-up period of 28.3 months (IQR:15–46 months) and a median number of 6 infusions (IQR:4–9) [109]. Notably, a subcutaneous formulation of ocrelizumab, branded as Ocrevus Zunovo, has been approved by the FDA for adults, with MS, offering an alternative route of administration that may enhance convenience and improve adherence.

While hypogammaglobulinemia was not seen frequently in the cohort described above, it is a well-documented concern with anti-CD20 therapies. A large retrospective study (n ​= ​468) of children and young adults treated with rituximab for various conditions found 23.2 ​% to have decreased IgG, and 40.8 ​% decreased IgM. Severe infections occurred in 17.9 ​% of patients, of which 3 were lethal. CD19^+^ or CD20^+^ cell numbers normalized a median of 9.0 months (IQR, 5.9–14.4 months) after discontinuation of therapy in the 135 patients in whom data regarding B cell counts following rituximab use were available [110]. Another multicenter study (n ​= ​144) treated with RTX for a variety of inflammatory disorders of the CNS reported that 7.6 ​% of pediatric patients experienced infections, with younger children (<5 years) being at higher risk for hypogammaglobulinemia [111].

Extended dosing intervals for B-cell depleting therapies (rituximab/ocrelizumab) have been proposed in POMS to try to mitigate risks related to immunosuppression. An observational pediatric cohort study comparing extended-interval dosing (n ​= ​12, median follow-up 18 months) to standard-interval dosing (n ​= ​9, median follow-up 6 months) of RTX/OCR found no confirmed disability worsening or new MRI lesions in either group. While over 87 ​% of the standard interval dosing group had infections vs. 38.5 ​% of the extended group, these differences were not statistically significant, potentially attributable to the small sample size [112]. Future studies are needed to better understand the long-term risks associated with B-cell depleting therapies in POMS.

3. Cladribine

Cladribine, a purine nucleoside analogue which leads to lymphocyte depletion, has been approved for the treatment of highly active relapsing-remitting multiple sclerosis (RRMS) in adults. It is administered for 4–5 consecutive days monthly for two months, with another course given the following year. In 2010, the CLARITY trial investigated the efficacy and safety of cladribine in adults with MS, at two different dosages (3.5 ​mg/kg/dose vs. 5.25 ​mg/kg/dose). Both dosage groups had a significant reduction in ARR (57.6 ​% decrease in the 3.5 ​mg/kg group and 54.5 ​% in the 5.25 ​mg/kg compared to placebo) as well as reduction in Gad-enhancing and active T2-weighted lesions [113]. As for POMS, data from both the international MSBase registry and Italian Multiple Sclerosis show that of the 104 patients (2.0 ​%) who received cladribine and only 3 received the medication before the age of 18 [114]. No granular data on the use of Cladribine in this age group is available, nor is data on dosing in patients <40 ​kg. In adult MS studies, cladribine has been associated with an increased risk of lymphopenia and infections. Cladribine is an appealing therapeutic option for select POMS cases due to its durable immunologic effects and long-term disease control. However, its use in children warrants caution because of the associated risk of malignancy.

4 Alemtuzumab

Alemtuzumab is a humanized monoclonal antibody targeting CD52, which is highly expressed on mature circulating B and T cells. The medication is administered intravenously for 5 consecutive days in two courses, with 12 months between each course. Trials in adult MS have shown alemtuzumab to have greater efficacy in reducing clinical relapses at year two compared to interferon-β-1a. Common adverse events include infusion-associated reactions, immune thrombocytopenia, and autoimmune thyroid diseases [115]. In POMS, clinical data on alemtuzumab remain limited. A Canadian group described two POMS patients who switched from teriflunomide to alemtuzumab. Both patients completed two infusions of alemtuzumab without experiencing any adverse events, and their disease remained stable during follow-up periods of 20 and 37 months, respectively [116]. Another group reported 10 POMS patients receiving alemtuzumab. One had a clinical relapse and four had radiological relapses after two infusions, with four needing additional infusions [117]. More recently, the LemKids phase 3 open-label study evaluated the safety, efficacy, and tolerability of alemtuzumab in pediatric patients with relapsing-remitting multiple sclerosis (RRMS). Eleven patients received alemtuzumab, and seven completed the treatment. Alemtuzumab treatment was associated with a lower number of new or enlarging T2 lesions compared with the prior-DMT phase (RR ​= ​0.04, 95 ​% CI: 0.01–0.14). Additionally, 64 ​% of participants remained relapse-free (ARR 0.18). No serious adverse events were reported, and safety profiles were consistent with those in adults. However, the study was prematurely terminated due to low enrollment, limiting the generalizability of its findings [118].

### Treatment approach

Two primary treatment strategies are employed in POMS (1) Stepwise Escalation and (2) induction using HET. The stepwise approach starts with low-to moderate-efficacy therapy, with escalation to HET in the presence of breakthrough disease. Patients may undergo horizontal (first-line therapies) or vertical switches (to a more potent therapy) based on disease activity. The second approach is an “induction” approach. This strategy starts with high-efficacy DMTs. Given evidence in the adult population as well as in POMS, outlined below, of improved disability outcomes with early initiation of 10.13039/501100000377DMT and use of HET in POMS, current practice favors the second approach [[119], [120], [121]]. Early initiation of disease-modifying therapy (DMT) in POMS is supported by studies which have demonstrated more relapses (higher ARR) with delayed treatment compared to those who started 10.13039/501100000377DMT within 6 months of their first event [[122], [123], [124], [125], [126]]. Furthermore, starting DMT more than two years post-diagnosis increases the risk of reaching sustained EDSS of 4 by 2.52-fold compared to initiating therapy within two years [123]. Moreover, observational data from the US Network of Pediatric MS Centers (n ​= ​741) demonstrated that patients on high-efficacy therapies (HETs) had significantly lower relapse rates (ARR 0.22 vs. 0.49, p ​< ​0.001) and a reduced risk of new/enlarging T2 lesions (HR ​= ​0.51, p ​< ​0.001) compared to those on injectable DMTs [127]. Similarly, longitudinal data from the Italian MS Registry and MSBase found that early HET initiation at minimal disability levels (EDSS 0–1.5) associated with lower risk of disability progression (HR 0.41, p ​< ​0.0001) over a median follow-up of 5.05 years [114]. Further supporting these findings, a French cohort study (n ​= ​64) reported that 90.9 ​% of pediatric patients on HETs remained relapse-free after 6.5 years, compared to 23.3 ​% on moderate-efficacy therapies (METs) [128].

There is mounting evidence that the use of early high-efficacy therapy (HET) in multiple sclerosis improves long-term outcomes, not only in terms of relapse prevention and disability, but also in preserving cognitive function [129]. A single-center observational study of 19 POMS patients provided early evidence of the cognitive benefit of HETs. Of the six patients who received early treatment with fingolimod or natalizumab, none developed cognitive impairment (CI) at last follow-up. In contrast, 69 ​% (9 of 13) of those not treated with HETs showed evidence for CI. These findings highlight the need for evaluation of cognitive outcomes in studies of therapies for POMS [130].

### Monitoring treatment response

Currently, there is no universally accepted definition of inadequate treatment response in POMS, but the concept of No Evidence of Disease Activity (NEDA)—defined as the absence of clinical and MRI disease activity – is of relevance for POMS as use of HETs has increased [[131], [132], [133]]. However, achieving NEDA is challenging, as longitudinal data from adult MS patients show that only 7 ​% maintain NEDA status at seven years of follow-up [134]. More recent iterations of NEDA, including NEDA-4 and NEDA-5 incorporate advanced MRI techniques and biomarkers such as NfL (discussed above) for improved disease monitoring, which are also of utility in POMS as noted above [135,136]. Notably, given the difficulty in attaining NEDA, Minimal Evidence of Disease Activity (MEDA) may be more attainable, although some studies suggest that MEDA is associated with faster disability accrual, necessitating ongoing refinement of treatment targets to ensure optimal long-term outcomes [137,138].

### Emerging therapies in POMS

Emerging therapies investigated in AOMS that have shown promising results include chimeric antigen receptor (CAR)-T therapy, immune reconstitution therapy (IRT), and Bruton's tyrosine kinase (BTK) inhibitors. To date, no studies have evaluated these therapies in pediatric-onset MS (POMS). Autologous hematopoietic stem cell transplantation (AHSCT) has also shown promise in refractory MS, with phase II trials in adults demonstrating reduced relapse rates and stabilized disability [[139], [140], [141]]. The use of AHSCT has not been described in POMS.

## Non-pharmacologic Interventions: Lifestyle Factors

### Diet

The role of diet in MS remains poorly understood, particularly in POMS. Studies in AOMS of the ketogenic diet that included adolescents with MS have demonstrated improvements in fatigue, quality of life, depression, and disability [142,143]. A multicenter study in the USA examined the associations between self-reported dietary intake and relapse rate in 219 POMS or clinically isolated syndrome (CIS) patients. The study found that each 10 ​% increase in energy intake from fat increased relapse risk by 56 ​% (HR 1.56, 95 ​% CI 1.05–2.31, p ​= ​0.027), each 10 ​% increase in saturated fat tripled relapse risk (HR 3.37, 95 ​% CI 1.34–8.43, p ​= ​0.009), while each additional one-cup equivalent of vegetable intake reduced relapse risk by 50 ​% (HR 0.50, 95 ​% CI 0.27–0.91, p ​= ​0.024) [144]. However, the study was limited by dietary data collection only at baseline and unmeasured confounders. There are no published studies of specific dietary interventions in youth with MS.

### Exercise

Exercise has been extensively studied in multiple sclerosis (MS), with a well-established body of evidence supporting its benefits in adults. Numerous studies have shown that aerobic exercise/physical activity (PA) is associated with improved fatigue, mood, mobility, and overall quality of life in adults with MS [[145], [146], [147], [148], [149], [150]]. In POMS, the data on exercise remain limited, but growing evidence suggests a positive association between physical activity levels and both disease and psychosocial outcomes in POMS. Children with MS tend to have lower physical fitness levels than healthy peers. Accelerometry data has demonstrated that youth with MS have fewer minutes per day of both moderate and vigorous PA than healthy peers (p ​= ​0.001). Notably, higher fitness levels correlated with lower disease activity in POMS [151,152]. Additionally, higher levels of PA correlate with lower disease activity, as demonstrated using MRI. A cross-sectional study of 110 POMS and CIS patients found that higher levels of vigorous physical activity correlated with lower MRT T2 lesion volumes (r ​= ​−0.66) and lower annualized relapse rate (r ​= ​−0.66). However, no significant association was observed between total brain volume and PA levels [153]. Beyond its impact on disease activity, higher levels of moderate-to-vigorous physical activity (MVPA) associates with lower levels of fatigue and depressive symptoms in POMS. A longitudinal evaluation has demonstrated that children with POMS with higher levels of MVPA had fewer depressive symptoms (p ​< ​0.001) and lower general fatigue scores than those with lower levels of MVPA through time (p ​< ​0.03) [154,155]. Recognizing the mental health benefits of exercise, tailored exercise programs have been developed, such as Active Teens with Multiple Sclerosis (ATOMIC), a mobile app promoting physical activity in POMS patients. A 2022 pilot study confirmed its feasibility, and a larger clinical trial is underway [156].

## **Symptomatic management**

Pain and fatigue are among the most prevalent and burdensome symptoms in POMS, whereas motor disability and spasticity are less commonly observed. Fatigue affects up to 43 ​% of children with MS and is frequently associated with anxiety, depression, and reduced quality of life [33,34,37]. Notably, a systematic review found that fatigue was not consistently related to clinical disease characteristics, neurocognitive functioning, or treatment status, but showed strong and consistent associations with depressed mood, reduced quality of life, and academic challenges [157].

As highlighted earlier, non-pharmacological strategies such as structured exercise programs have demonstrated benefits in alleviating fatigue [154,155]. Additional lifestyle modifications including dietary changes, vitamin D supplementation, heat avoidance, and cooling strategies may also contribute to symptom relief. A recent phase II study demonstrated that a monitored ketogenic diet was safe, well tolerated, and associated with significant improvements in fatigue, depression, body composition, and overall quality of life in individuals with relapsing MS, including pediatric participants [143].

Pharmacologic treatments for fatigue have primarily been studied in adults. Modafinil, a stimulant approved for narcolepsy, and amantadine have shown some benefit in earlier trials using the Modified Fatigue Impact Scale [158,159]. However, a more recent phase III randomized, placebo-controlled, crossover trial comparing modafinil, amantadine, and methylphenidate found that none of the three agents were superior to placebo in reducing fatigue, and all were associated with a higher frequency of adverse events [160]. Psychotherapy and counseling may play a valuable role in addressing mood-related fatigue and neuropsychiatric comorbidities in children with MS.

In parallel, pain is now acknowledged as a significant symptom in pediatric MS. A recent Delphi consensus estimated that recurrent, MS-related pain affects 25–50 ​% of children. Reported pain types include neuropathic pain, spasticity-related pain, and various forms of headache. Interim guidelines recommend routine pain assessment using developmentally appropriate tools and advocate for a multidisciplinary approach that combines pharmacologic and psychosocial interventions [161]. Early recognition and comprehensive care are essential to effectively manage pain and improve quality of life in children living with MS.

## Conclusion

Children with MS have high levels of disease activity, fatigue, and depression. Real-world studies have demonstrated clear benefits to early treatment with HET in POMS. These studies have shown that most therapies currently approved for use in adults with MS can be used safely in POMS. However, the management of pediatric-onset multiple sclerosis (POMS) presents challenges due to the limited number of approved therapies, thus limiting access to these important therapies in children with MS. Inclusion of pediatric patients in clinical trials for MS therapies is therefore a critical future need.

High levels of fatigue and depression in POMS, which persist despite the use of HET, highlight the need for attention to interventions targeting these problems. Promising studies in adults with MS suggest that specific dietary interventions, such as the ketogenic diet, may have benefits for these outcomes. Other studies have pointed to associations between higher levels of vigorous physical activity and lower levels of disease activity, fatigue and depression in POMS. Further research on lifestyle interventions focused on diet and physical activity in POMS is needed. These interventions may be pivotal to improving the quality of life and prognosis of children with MS.

## Author contributions

L.A. conducted the literature search, performed the literature review and drafted the manuscript. E.A.Y. provided guidance throughout the process and critically revised the manuscript. Both authors have read and approved the final submitted manuscript and agree to be accountable for the work.

## Funding

E.A. Yeh has received research funding from NMSS, CMSC, CIHR, NIH, OIRM, SCN, CBMH Chase an Idea, SickKids Foundation, Rare Diseases Foundation, MS Scientific Foundation, McLaughlin Centre, Leong Center, Peterson Foundation, Gary Hurvitz Centre for Brain and Mental Health. Investigator initiated research funding from Biogen. Scientific advisory: Hoffman-LaRoche, Alexion. DSMB: Pipeline Therapeutics. Speaker honoraria: Biogen, JHU, Saudi Epilepsy Society, NYU, MS-ATL; ACRS, PRIME, CNPS. Co-Editor in Chief, MSARD. Governing Council: CANTRAIN. Steering Committee: Rare-Kids CAN.

## Declaration of competing interest

The authors declare that they have no known competing financial interests or personal relationships that could have appeared to influence the work reported in this paper.
